# Effects of Popular Diets without Specific Calorie Targets on Weight Loss Outcomes: Systematic Review of Findings from Clinical Trials

**DOI:** 10.3390/nu9080822

**Published:** 2017-07-31

**Authors:** Stephen D. Anton, Azumi Hida, Kacey Heekin, Kristen Sowalsky, Christy Karabetian, Heather Mutchie, Christiaan Leeuwenburgh, Todd M. Manini, Tracey E. Barnett

**Affiliations:** 1 Department of Aging and Geriatric Research, University of Florida, Gainesville, FL 32611, USA; kaceydheekin@ufl.edu (K.H.); ckarabetian@phhp.ufl.edu (C.K.); heather.mutchie@som.umaryland.edu (H.M.); cleeuwen@ufl.edu (C.L.); tmanini@ufl.edu (T.M.M.); 2 Department of Clinical and Health Psychology, University of Florida, Gainesville, FL 32611, USA; 3 Department of Nutritional Science, Faculty of Applied Bioscience, Tokyo University of Agriculture, Tokyo 1568502, Japan; 4 Department of Applied Physiology and Kinesiology, University of Florida, Gainesville, FL 32611, USA; ksowalsky@ufl.edu; 5 Department of Gerontology, University of Maryland, Baltimore, MD 21201, USA; 6 Department of Health Behavior and Health Systems, School of Public Health, University of North Texas Health Science Center, 3500 Camp Bowie Dr., Fort Worth, TX 76107, USA; Tracey.Barnett@unthsc.edu

**Keywords:** carbohydrates, protein, body composition, obesity, overweight

## Abstract

The present review examined the evidence base for current popular diets, as listed in the 2016 U.S. News & World Report, on short-term (≤six months) and long-term (≥one year) weight loss outcomes in overweight and obese adults. For the present review, all diets in the 2016 U.S. News & World Report Rankings for “Best Weight-Loss Diets”, which did not involve specific calorie targets, meal replacements, supplementation with commercial products, and/or were not categorized as “low-calorie” diets were examined. Of the 38 popular diets listed in the U.S. News & World Report, 20 met our pre-defined criteria. Literature searches were conducted through PubMed, Cochrane Library, and Web of Science using preset key terms to identify all relevant clinical trials for these 20 diets. A total of 16 articles were identified which reported findings of clinical trials for seven of these 20 diets: (1) Atkins; (2) Dietary Approaches to Stop Hypertension (DASH); (3) Glycemic-Index; (4) Mediterranean; (5) Ornish; (6) Paleolithic; and (7) Zone. Of the diets evaluated, the Atkins Diet showed the most evidence in producing clinically meaningful short-term (≤six months) and long-term (≥one-year) weight loss. Other popular diets may be equally or even more effective at producing weight loss, but this is unknown at the present time since there is a paucity of studies on these diets.

## 1. Introduction

Against the backdrop of the obesity epidemic and the inability of most individuals to sustain weight loss induced by calorie-restricted diets [1], alternative dietary approaches to achieve short- and long-term weight loss have become of increasing scientific interest [2]. Up until recently (2015), the Dietary Guidelines for Americans recommended that macronutrient intake consist of 45–65% of daily energy intake from carbohydrates, 20–35% from fats, and 10–35% from protein [3]. In line with these recommendations, the results of the U.S. National Health and Nutrition Examination Survey (NHANES) showed that carbohydrate consumption increased from 39% of total energy intake in 1971 to 51% in 2011. During this same time period, however, the percentage of overweight Americans also increased dramatically (from 42% to 66%) [4]. Based in part on such trends in weight gain, the creators of many popular diets (e.g., Atkins, Zone) have suggested that diets in which carbohydrate intake is significantly higher than other macronutrients are not an optimal approach for weight loss and may even contribute to weight gain. Most of these diets are published and promoted by one or more health and wellness “experts” who attest to the health and weight loss benefits observed when following their recommended diet.

Despite their popularity among the general public, the efficacy of many popular diets for weight loss has been called into question by researchers, nutrition experts, and health care professionals [5,6,7]. A meta-analysis by Johnston et al. (2014) previously attempted to answer the question of whether any popular diets were effective in producing weight loss over the short term (six months or less) and/or long term (12 months) [2]. The primary findings of this meta-analysis were that reductions in calorie intake were the primary driver of weight loss and that differences between diets differing in macronutrient composition were relatively small.

Although the findings of the Johnson et al. (2014) meta-analysis are of high importance, a potential factor confounding the interpretation of these findings was that this review included studies in which participants were specifically instructed to reduce their caloric intake and/or increase physical activity levels, beyond the recommendation of the popular diet [2]. To our knowledge, the effectiveness of specific popular diets on weight loss outcomes in dietary interventions that did not include specific calorie targets and/or structured (i.e., supervised) physical activity recommendations has not been examined. Therefore, the purpose of our review was to examine the effects of the most widely recognized popular diets of 2016, in their proposed format, on both short- and long-term weight loss outcomes in overweight and obese individuals, based on findings from clinical trials that did not include specific calorie targets, meal replacements, supplementation with commercial products, and/or structured exercise programs.

## 2. Materials and Methods

This systematic review followed the Preferred Reporting Items for Systematic Review and Meta-Analysis (PRISMA) guidelines and the protocol was prospectively registered with Prospective Register for Systematic Reviews (PROSPERO; registration number: CRD42017056770). The 2016 U.S. News & World Report Rankings for “Best Weight-Loss Diets” listed and evaluated 38 popular diets. According to the U.S. News & World Report’s “Best Diet” methodology, a panel of experts examined the research regarding each diet’s potential to produce both short-term and long-term weight loss and assigned scores and ratings accordingly. For the present review, all diets in the 2016 U.S. News & World Report rankings for “Best Weight-Loss Diets”, which did not involve specific calorie targets, meal replacements, supplementation with commercial products, and/or were not categorized as “low-calorie” diets were examined. Based on these criteria, a total of 20 out of 38 diets were included in this review. All 38 diets are summarized according to diet type in Table 1. More information on the theories and guidelines of each of the eligible 20 popular diets is available online in the Health section of the U.S. News & World Report’s website under “Best Diets”.

### 2.1. Data Sources

Literature searches were conducted by two separate individuals on each of the 20 eligible diets on PubMed through the National Center of Biotechnology Information, The Cochrane Database of Systematic Reviews (CDSR), the Cochrane Central Register of Controlled Trials (CENTRAL), and Web of Science until September 2016.

### 2.2. Inclusion Criteria

The search terms included the diet name (e.g., “Atkins Diet”), “overweight or obesity”, and the word “weight”. Through PubMed, filters were set to allow only “clinical trials,” “human” studies, and “English” studies to be displayed between 1980 and September 2016. Our preset inclusion criteria were the following: (1) interventional clinical trials; (2) sample sizes of at least 15 per group; (3) intervention periods of 12 weeks or longer; (4) inclusion of adult participants (>18 years) with a body mass index (BMI) ≥25 kg/m^2^; (5) objective measures of body weight pre- and post-intervention; and (6) articles written in the English language. For the purposes of this review, clinical trials of between three and six months in duration were considered short-term, and clinical trials one year or longer were considered long-term (no studies were longer than six months but less than one year). Clinically meaningful or “successful” weight loss was defined as weight loss that was equivalent to 5% or more of participants’ baseline weight [17,18]. All identified clinical trials met our short- and long-term criteria listed above.

### 2.3. Exclusion Criteria

Each dietary intervention had to exclusively follow the dietary guidelines of the diet of interest. If the participants were provided with a specific menu or modified version of the diet, then these studies were excluded. Clinical trials with dietary interventions that had explicit calorie targets and/or structured physical activity components were excluded. We chose not to include these studies because specific caloric targets and/or supervised exercise programs are likely to produce weight loss and thereby confound potential effects of popular diets on weight loss outcomes.

### 2.4. PubMed Search and Study Selection

**Figure 1:**
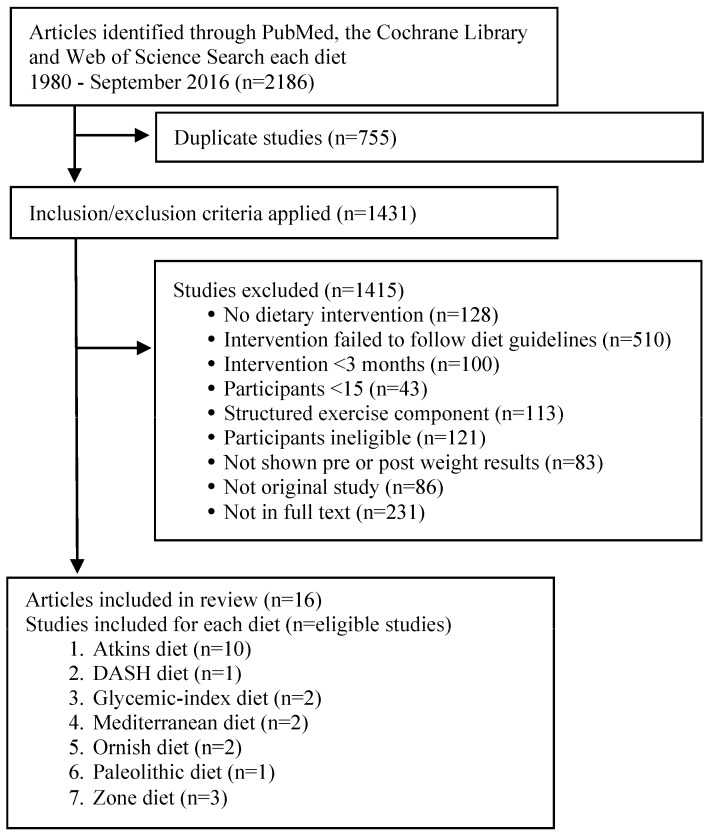
The Preferred Reporting Items for Systematic Review and Meta-Analysis (PRISMA) flow diagram of the literature search results. DASH, Dietary Approaches to Stop Hypertension.

The PubMed search was conducted in the following manner: (each diet name) AND (weight OR (body mass) OR (body mass index)) AND (overweight OR obesity). Limits were humans, clinical trial, adult, and English. The preferred Reporting Items for Systematic Reviews diagram displaying the process flow is presented in Figure 1.

Three authors (A.H., K.H. and H.M.) extracted data from each paper independently and compared their findings for discrepancies. Any discrepancies were reviewed and resolved by a senior author (SDA). All authors reviewed final candidate papers to verify and agree that they met the inclusion criteria.

### 2.5. Data Extraction

The mean weight change and 95% confidence interval (CI) for the findings of each eligible study were extracted for this review. A number of studies reported only the baseline and post-treatment means. In these cases, estimated standard deviation and 95% CI were calculated (Supplementary Materials).

### 2.6. Assessing Risk of Bias

Three authors (A.H., K.H. and H.M.) independently assessed each study for the assignment of ratings of low, unclear, or high risk of bias related to the selection, performance, detection, attrition, reporting and other potential sources of bias by using the Cochrane Collaboration Handbook guidelines [19]. Items that were not rated the same were discussed until a consensus was reached.

## 3. Results

### 3.1. Study Selection

Using the systematic search terms listed above with the limited filter criteria, 1633 articles were identified. Figure 1 displays a flow diagram of the literature search results. However, relatively few of these studies met all of the preset inclusion criteria. The most common factors for exclusion were the following: (1) study did not have a dietary intervention; (2) intervention failed to follow diet guidelines (e.g., the intervention had explicit calorie restriction guidelines/specific calorie targets or changed the macronutrient guidelines or the established guidelines of the diet of interest); (3) limited length of dietary interventions; (4) ineligible participant demographics (e.g., participants’ BMI <25 kg/m^2^, <18 years of age, disease); (5) small sample sizes (*n* <15); (6) intervention had structured physical activity/exercise components; (7) article did not report weight changes; (8) article was not the original study; and (9) there was not a full-text article available.

### 3.2. Study Characteristics

There were a higher proportion of women than men enrolled in most of the studies (Table S1). The age ranged between 18 and 70 years, and the BMI ranged between 25 and 44 kg/m^2^.

### 3.3. Risk of Bias

A couple of studies did not report the recruitment and randomization process, but simply stated that the participants were randomized (Table S2). All studies specified the eligibility criteria. Participant completion rates varied widely across studies (range = 16% to 97%). A couple of articles did not use the intention to treat analysis, and thus the attrition bias in these studies is unclear. Another source of potential bias is that most studies did not include a control diet.

### 3.4. Main Findings

**Figure 2:**
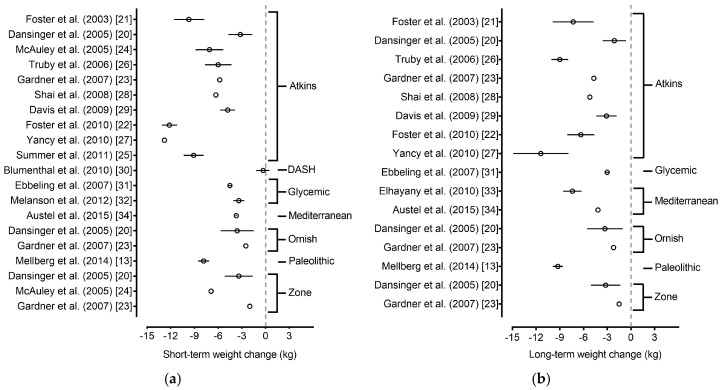
Forest plot of short-term and long-term weight loss (absolute body mass change) among eligible diets. Forest plot depicting (**a**) short-term and (**b**) long-term weight loss outcomes in overweight and obese adults among eligible popular diets from the 2016 U.S. News & World Report. Values are shown as mean differences and 95% confidence interval. The eligible diets consisted of the following: Atkins, DASH, Glycemic-Index, Mediterranean, Ornish, Paleolithic and Zone diets. Note: Long-term results from Truby et al. are a follow-up of nine participants who voluntarily followed the Atkins diet after completing the initial six-month intervention [26].

Clinical trials that met our preset criteria listed above were available for 7 of the 20 eligible popular diets. These diets included: (1) Atkins Diet; (2) DASH Diet; (3) Glycemic-Index Diet; (4) Mediterranean Diet; (5) Ornish Diet; (6) Paleolithic Diet; and (7) Zone Diet. Of those diets, The Atkins, Glycemic index, Mediterranean, Ornish, and Zone diets were tested in at least two clinical trials that met our predefined criteria. The number of eligible clinical trials identified for these diets ranged from one to 10 clinical trials per diet. The findings from clinical trials conducted on all seven of the diets are outlined in Figure 2 and Table 2 [13,20,21,22,23,24,25,26,27,28,29,30,31,32,33,34].

#### 3.4.1. Atkins Diet

Ten clinical trials, ranging in duration from three months to 24 months, were identified for the Atkins Diet [20,21,22,23,24,25,26,27,28,29]. For brevity and clarity, the findings from all 10 of these clinical trials are summarized in Figure 2a,b. Nine of the 10 clinical trials supported the ability of the Atkins Diet to produce clinically meaningful short-term weight loss, and six of the eight long-term clinical trials supported the effectiveness of this diet for long-term weight loss.

#### 3.4.2. Dietary Approaches to Stop Hypertension (DASH) Diet

One short-term clinical trial was available for the DASH Diet [30]. In this clinical trial, conducted by Blumenthal et al. (2010), 46 overweight and obese adults (age ≥ 35 years and BMI 25.0–40.0 kg/m^2^) with high blood pressure were randomized to the DASH Diet alone or the DASH diet with aerobic exercise and caloric restriction for a four-month time period. After four months, participants in the DASH Diet alone group maintained their weight, with an average weight change of 0.3% (−0.3 kg, 95% CI: −1.2 to 0.5 kg). Participants’ dietary energy was 19.4% protein, 53.8% carbohydrate, and 27.8% fat.

#### 3.4.3. Glycemic-Index Diet

Two short-term and one long-term clinical trials were available for the Glycemic Index Diet. Ebbeling et al. (2007) examined 36 obese young adults (age 18–35 years old and BMI ≥30 kg/m^2^) assigned to a low glycemic-index diet for 18 months and found that the low glycemic-index diet produced an average weight loss of 4.3% (4.5 kg) after six months, and −2.9% (3.0 kg) after 12 months [31]. Melanson et al. (2012) examined 59 sedentary, overweight, and obese adults (aged 25–50 years and BMI = 27.0–35.0 kg/m^2^) assigned to a low glycemic index diet for three months and found that the low glycemic index diet produced an average weight loss of 4.0% (3.4 kg) during this time period [32].

#### 3.4.4. Mediterranean Diet

One short- and two long-term clinical trials were available for the Mediterranean Diet [33,34]. Elhayany et al. (2010) conducted a study in which 89 overweight and obese diabetic adults (aged 30–65 years and BMI = 27.0–34.0 kg/m^2^) were randomized to a traditional Mediterranean Diet, a low carbohydrate Mediterranean Diet, or the 2003 American Diabetic Association (ADA) Diet for 12 months [33]. After 12 months, the traditional Mediterranean Diet produced an average weight loss of 8.7% (7.4 kg) and the low-carbohydrate Mediterranean Diet produced an average weight loss of 10.3% (8.9 kg). Additionally, Austel et al. (2015) conducted a study in which 100 overweight and obese adults (aged 52.4 ± 0.9 years, BMI = 30.1 ± 0.3 kg/m^2^) were randomized to follow the Mediterranean Diet for a one-year period [34]. Participants’ mean weight loss was 7.2% (6.1 kg) after three months and 4.9% (4.2 kg) after 12 months. 

#### 3.4.5. Ornish Diet

Two short- and long-term clinical trials were available for the Ornish Diet [20,23]. Dansinger et al. (2005) conducted a study in which 40 overweight and obese adults between the ages of 22 and 72 years with known hypertension, dyslipidemia, or fasting hyperglycemia were assigned to the Ornish Diet for 12 months [20]. In this study, the Ornish Diet produced an average weight loss of 3.5% (3.6 kg) after six months and 3.2% (3.3 kg) after 12 months. Gardner et al. (2007) conducted a study in which 311 overweight and obese premenopausal women between the ages of 25 and 50 years were randomized to the Ornish Diet (*n* = 76), Atkins Diet (*n* = 77), Zone Diet (*n* = 79), or LEARN (Lifestyle, Exercise, Attitudes, Relationships, and Nutrition) Diet (*n* = 79) for 12 months [23]. The Ornish Diet produced an average weight loss of approximately 2.9% (2.4 kg) after six months (based on chart analysis) and 2.6% (2.2 kg, 95% CI: −3.6 to −0.8 kg) after 12 months.

#### 3.4.6. Paleolithic Diet

One short- and one long-term clinical trial were available for the Paleolithic Diet [13]. Mellberg et al. (2014) conducted a study in which 27 overweight and obese postmenopausal women (aged 59.5 ± 5.5 years and BMI ≥27.0 kg/m^2^) were randomized to the Paleolithic Diet for 24 months. The Paleolithic Diet provided 30% of daily energy intake from protein, 40% from fats, and 30% from carbohydrates. The Paleolithic Diet produced an average weight loss of 9.0% (7.9 kg) after six months and 10.6% (9.2 kg) after 12 months. 

#### 3.4.7. Zone Diet

One short-term and two long-term clinical trials were available for the Zone Diet [20,23,24], which recommends that 30% of calories come from protein, 30% from fats, and 40% from carbohydrates. McAuley et al. [24] conducted a study in which 30 overweight and obese women (aged between 30–70 years and BMI ≥ 27.0 kg/m^2^) were randomized to the Zone Diet for six months. After six months, participants in the Zone Diet group lost approximately 7.4% (6.9 kg) of their baseline weight. Dansinger et al. conducted a study in which 40 overweight and obese adults were randomized to the Zone Diet [20]. In this study, participants had mean weight losses of 3.4% (3.4 kg) after six months and 3.2% (3.2 kg) after 12 months. In a study conducted by Gardner et al. [23], 79 overweight and obese premenopausal women achieved mean weight losses of 2.4% (2.0 kg) after six months and 1.8% (1.5 kg) after 12 months.

## 4. Discussion

The purpose of this review was to examine the clinical evidence supporting the effectiveness of current popular diets that did not include specific calorie targets, meal replacements, supplementation with commercial products, and/or structured exercise programs on both short-term (≤six months) and long-term (≥one year) weight loss outcomes. There were a number of important findings of this review. First, clinical trials that tested popular diets as recommended (without specific calorie targets) were available for only seven of the 20 eligible popular diets in the 2016 U.S. News & World Report. This indicates that the majority of popular diets have not been rigorously empirically tested in human clinical trials as they are currently recommended. Thus, it is difficult to evaluate the efficacy of the vast majority of popular diets based on evidence from clinical trials at the present time. Second, there was a large disparity in the evidence base for these seven diets, with the Atkins Diet having substantially more support than the other seven empirically tested diets (i.e., the DASH Diet, the Glycemic-Index Diet, the Mediterranean Diet, the Ornish Diet, the Paleolithic Diet, and the Zone Diet). Specifically, findings from nine of 10 clinical trials supported the efficacy of the Atkins Diet in producing clinically meaningful short-term weight loss, with findings from six of eight trials supporting the ability of this diet to produce long-term weight loss.

The findings of this review are not in line with current recommendations of the Dietary Guidelines Advisory Committee, which state that diets with less than 45% of calories as carbohydrates are not more successful than other diets for long-term weight loss (12 months) [35]. As noted above, we found that the Atkins Diet produced substantial long-term weight losses in a number of clinical trials [20,21,22,23,24,25,26,27,28,29]. Additionally, the Paleolithic diet, another diet that advocates less than 45% of calories being consumed as carbohydrates, was also found to produce substantial short- and long-term weight loss in a recent clinical trial [13]. Although we found diets with low carbohydrate content to be effective at producing short- and long-term weight loss, the safety of this dietary approach needs to be critically examined [36,37].

When considering the findings of this review, it is important to remember that successful clinical weight loss was reported according to generally accepted criteria for clinically meaningful weight loss (≥5% body weight) in overweight and obese adults instead of significant weight change from baseline [38]. Weight losses of this magnitude have been found to produce beneficial changes in blood pressure, blood glucose, lipid profiles, and psychological well-being [39]. Noteworthy, lifestyle interventions involving caloric restriction typically produce mean weight losses of 5 to 10 kg over the course of four to six months [40]. Thus, the magnitude of weight loss achieved by the popular diets is in line with that typically achieved for calorie-restricted diets.

Although a recent meta-analysis by Johnston et al. (2014) used similar criteria to define clinically meaningful weight loss, our findings differed from their review which concluded that “These findings support recent recommendations for weight loss in that most calorie-reducing diets result in clinically important weight loss as long as the diet is maintained [2]”. In contrast, the findings of our review indicated that clinically meaningful short- and long-term weight loss can be achieved without restricting calories per se but rather by following the recommendations of some popular diets. One likely reason for the discrepancy in findings is the difference in eligibility criteria used to select studies. In contrast to the Johnston et al. [2] meta-analysis, studies in which the dietary interventions incorporated specific calorie and/or exercise recommendations were not included in the present review. We chose not to include these studies because specific caloric targets and/or supervised exercise programs are likely to produce weight loss and thereby confound potential effects of popular diets on weight loss outcomes. Additionally, the majority of popular diets do not include specific caloric recommendations (Table 1), so individuals following these diets would not typically set caloric intake goals.

A critical question related to which popular diet is the most effective for producing weight loss is, “*What are the potential mechanisms through which the popular diets promote weight loss?*” Some diet advocates (e.g., Atkins Diet) assert that limiting carbohydrate consumption is the primary driver of weight loss [40], while others argue that restriction of specific macronutrients can lead to a reduction in total calorie intake, and that calorie restriction is the primary driver of weight loss. Although it is clear that calorie restriction produces short-term weight loss, a growing body of research supports low-carbohydrate, high fat dietary approaches for healthy weight management [41]. These findings have led to increasing interest regarding the potential mechanisms through which dietary macronutrient content may promote or discourage weight loss. For example, Ebbeling et al. (2012) demonstrated that following weight loss, low-fat, high carbohydrate diets produced greater reductions in resting and total energy expenditure than other diets, whereas diets with low-carbohydrate and higher fat content produced the smallest reductions in energy expenditure during isocaloric feeding following weight loss [42]. In line with the findings of Ebbeling et al. (2012), the findings of the present review suggest that high fat, low carbohydrate diets are most advantageous for promoting long-term weight loss.

There are several limitations to the present review. First, there were a limited number of clinical trials available from which to evaluate weight loss outcomes of popular diets that did not have specific calorie targets or structured exercise programs. Due to the limited number of published studies, we were not able to statistically compare weight loss differences between individual diets. The small number of clinical trials examining the efficacy of many popular diets is concerning, as it indicates relatively little empirical evidence exists to support many current popular diets available, which are heavily marketed to the public.

A second limitation is that our analyses were based only on the randomized dietary assignment and did not account for adherence to the actual macronutrient composition of the specific diet. Unfortunately, there is a lack of information on adherence to popular diets as well as weight loss outcomes. In a few of the studies included in this review, attrition levels were high (>40%), which suggests individuals had trouble adhering to the diet. For example, long-term results of the study conducted by Truby and colleagues were based on a 12-month follow-up of only nine out of 57 randomized participants who volitionally chose to adhere to the Atkins diet after completing the initial six-month intervention [26]. It is noteworthy that only a small percentage (16%) of the individuals in the randomized sample chose to remain on the diet following the intervention.

Another limitation of the review is that we reported all weight changes as weight change from baseline rather than as a difference from a control group. An additional limitation is that weight loss was the only outcome included in this review. Changes in waist circumference, BMI, and body composition might provide more evidence from which to evaluate the efficacy of these diets. Additionally, assessment of the effects of popular diets on cardiovascular, metabolic (e.g., blood pressure and serum lipid concentrations), and functional outcomes could reveal information on the safety of these diets. During aging, there is typically an increase in body fat mass and a corresponding loss of muscle mass and strength [43,44]. In line with this, individuals who are “normal weight” or “healthy weight” but have a high body fat percentage have recently been recognized as an at risk group, [45] as they often show signs of metabolic dysregulation normally associated with obesity [46,47] and have increased risk of cardiovascular disease [48,49]. Such findings highlight the importance of assessing body composition and not just body mass (weight) in future weight loss intervention trials.

This review also had a number of strengths. First, to our knowledge, the present review is the first to compile the findings from clinical trials that objectively measured weight loss associated with popular diets in the absence of explicit calorie restriction targets and/or structured exercise components. Additionally, the inclusion criteria for the clinical trials in this review were rigorous. The reason for such explicit criteria was to ensure that only methodologically-strong studies were included. However, our study eligibility criteria may have eliminated some studies and clinical evidence that might provide a broader perspective on the differences between the popular diets on weight loss outcomes.

## 5. Conclusions

In conclusion, the findings of the present review indicate that of all the current popular diets, the Atkins Diet was tested in the greatest number of clinical trials and had the most evidence in producing clinically meaningful short-term (≤six months) and long-term (≥one year) weight loss. There was limited evidence supporting the effectiveness of other popular diets in producing clinically meaning short- and long-term weight loss. Thus, more comparative evidence is needed in order to better evaluate the efficacy of each of these popular diets in promoting both short- and long-term weight loss.

## Figures and Tables

**Table 1:** Summary of 2016 U.S. News & World Report “Best Diets” considered for literature review.

Diet Name	Diet Type (Macronutrient Composition)	Calorie-Specific Recommendation	Exercise Component
Abs Diet	6 meals/day, emphasis on protein	None	Required
Acid Alkaline Diet	80% high pH (7–14) foods, 20% low pH (0–7) foods	None	Not Specified
Anti-Inflammatory Diet	Healthy fats ^d^, complex carbs and limited animal protein	2000–3000 kcal/day ^c^	Encouraged
Atkins Diet [8]	During the first 2 weeks, less than 20 g of carbohydrate daily, with a gradual increase to 50 g daily	None	Encouraged
Biggest Loser Diet	Emphasis on complex carbs, lean proteins, few saturated fats and sugars	None	Required
Body Reset Diet ^a^	Low-calorie, plant-based diet, mostly smoothies for 2 weeks	None	Required
DASH Diet ^b^ [3,9]	Emphasis on complex carbs, lean protein, low-fat dairy, fruits and vegetables	Monitored ^c^	Encouraged
Dukan Diet	High-Protein, low-fat, low-carb	None	Required
Eco-Atkins Diet [10]	Low-Carb & exclusion of animal proteins	None	Not Specified
Engine 2 Diet	Vegan diet with no vegetable oils	None	Encouraged
Flat Belly Diet	Plant-based fats in every meal; complex carbs, lean protein and healthy fats ^d^	1600 kcal/day ^c^	Encouraged
Flexitarian Diet	Mostly vegetarian, utilizing animal proteins sparingly	1500 kcal/day	Encouraged
Glycemic-Index Diet [11]	Mostly low GI (≤55), some medium GI (56–69) and few high GI (≥70) foods	None	Not Specified
HMR Diet ^a^	Meal Replacement	None	Encouraged
Jenny Craig Diet ^a^	Meal Replacement	1200–2300 kcal/day ^c^	Required
Macrobiotic Diet	Emphasis on whole “living” foods: vegetarian and organic	None	Encouraged
Mayo Clinic Diet	Emphasis on complex carbs, low in saturated fat and salt	None	Required
Medifast Diet ^a^	Meal Replacement	800–1000 kcal/day	Encouraged
Mediterranean Diet [3]	Complex carbs and healthy fats ^d^; few red meats, sugars and saturated fats	None	Required
MIND Diet	Emphasis on vegetables, nuts, berries, beans, whole grains, fish, poultry and olive oil (DASH + Mediterranean Diet)	None	Not Specified
Nutrisystem Diet ^a^	Meal Replacement	None	Encouraged
Ornish Diet [12]	A vegetarian diet containing 10% of calories from fat	None	Encouraged
Paleolithic Diet [13]	Focus on meats, fruits and vegetables; cuts out refined sugar, diary and grains	None	Encouraged
Raw Food Die t ^a^	75–80% plant based foods; all food is never heated over 115 °F	None	Not Specified
Slim-Fast ^a^	Meal Replacement	1200 kcal/day	Encouraged
South Beach Diet	Low-carb, high-protein and healthy fats ^d^	None	Required
Spark Solution Diet	Balanced (45–65% carbs, 20–35% fats and 16–35% proteins)	≤1500 kcal/day	Required
Supercharged Hormone Diet	2-week detox to identify and remove allergenic/inflammatory food	None	Required
The Fast Diet ^a^	5 days of normal meals, 2 non-consecutive days of fasting	M, 600 kcal/day; F, 500 kcal/day (2 day/week)	Not Specified
The Fertility Diet	Emphasis on plant proteins, whole grains	None	Encouraged
TLC Diet	Low-Fat; no more than 200 mg dietary cholesterol daily, red meat discouraged	M, 1600–2500 kcal/day; F, 1200–1600 kcal/day	Required
Traditional Asian Diet	Low-fat, emphasis on rice, vegetables, fresh fruit, fish; red meat sparingly	None	Not Specified
Vegan Diet [14]	Exclusion of all animal products and bi-products	None	Not Specified
Vegetarian Diet [3]	Exclusion of animal proteins	None	Not Specified
Volumetrics Diet	Emphasis on low calorie, high volume foods	None	Encouraged
Weight Watchers^®^	Point values based on macronutrient composition, personalized point cap/day	None	Encouraged
Whole30 Diet	Avoid sugar, alcohol, grains, dairy, and legumes for 30 days	None	Not Specified
Zone Diet ^b^ [15]	Balanced (40% carbs, 30% protein, and 30% fat)	M, 1500 kcal/day; F, 1200 kcal/day	Encouraged

The diets listed in this table were from the Best Weight-Loss Diets Ranking website [16]. Note: carb(s) = carbohydrate; DASH = Dietary Approaches to Stop Hypertension. HMR = Health Management Resources; MIND = Mediterranean-DASH Intervention for Neurodegenerative Delay; TLC = Therapeutic Lifestyle Changes. ^a^ Diet excluded from literature review due to low caloric intake, commercial meal replacement component(s), and/or specific caloric limits. ^b^ Diets included in literature review, due to no specified caloric limit in clinical studies, although defined by U.S. News & World Report to include caloric limit. ^c^ Caloric restriction based on demographic factors such as age, gender, activity level, and/or current weight. ^d^ Healthy fats may refer to monounsaturated fat, polyunsaturated fat, and/or omega-3 fatty acids.

**Table 2:** Short- and long-term weight loss outcomes among eligible diets.

Diet Reference	*N*	Age (Years)	Baseline BMI (kg/m^2^)	Short-Term			Long-Term			Macronutrients Protein:Fat:Carbohydrate (%)
				Period (Mo)	Weight Change		Period (Mo)	Weight Change		
					(kg)	(%)		(kg)	(%)	
**Atkins**										
Foster et al. [21]	33	44 ± 9.4	33.9 ± 3.8	3	−8.0	−8.1	12	−7.2	−7.3	
				6	−9.6	−9.7				
Dansinger et al. [20]	40	47 ± 12	35 ± 3.5	6	−3.2 ± 4.9	−3.5	12	−2.1 ± 4.8	−2.1	18:37:50 (Base)
										26:50:16 (1 Mo)
										18:39:41 (6 Mo)
										18:38:40 (12 Mo)
McAuley et al. [24]	31	45 ± 7.4	36.0 ± 3.9	4	−6.9	−7.2	No data			18:34:44 (Base)
										29:57:11 (2 Mo)
				6	−7.1	−7.4				24:47:26 (6 Mo)
Truby et al. [26]	57	40.9 ± 9.7	31.9 ± 2.2	6	−6.0 ± 6.4	−6.6	12 *	−9.0 ± 4.1 *	−10.0	
Gardner et al. [23]	77	42 ± 6	32 ± 4	6	−5.8	−6.7	12	−4.7	−5.5	17:36:46 (Base)
										28:55:18 (2 Mo)
										22:47:30 (6 Mo)
										21:44:35 (12 Mo)
Shai et al. [28]	109	52 ± 7	30.8 ± 3.5	6	−6.3	−6.9	12	−5.2	−5.7	19:31:51 (Base)
										22:39:41 (6 Mo)
							24	−4.7	−5.1	22:39:42 (12 Mo)
										22:39:40 (24 Mo)
Davis et al. [29]	55	54 ± 6	35 ± 6	3	−5.2	−5.6	12	−3.1	−3.3	20:36:44 (Base)
										23:43:34 (6 Mo)
				6	−4.8	−5.1				23:44:33 (12 Mo)
Foster et al. [22]	153	46.2 ± 9.2	36.1 ± 3.6	6	−12.2	−11.8	12	−10.9	−10.5	
							24	−6.3	−6.1	
Yancy et al. [27]	72	52.9 ± 10.2	39.9 ± 6.9	3	−9.7	−7.9	12	−11.4	−9.2	16:40:44 (Base)
										30:59:10 (2 w)
										29:57:12 (3 Mo)
				6	−12.8	−10.4				28:57:13 (6 Mo)
										26:57:15 (12 Mo)
Summer et al. [25] ^†^	42	44.5 ± 9.2	33.2 ± 2.6	4/6	−9.1	−10.1	No data			16:36:48 (Base)
										24:49:27 (Post-intervention)
**DASH**										
Blumenthal et al. [30]	46	51.8 ± 10	32.8 ± 3.4	4	−0.3	−0.3	No data			
**Glycemic Index**										
Ebbling et al. [31]	36	28.2 ± 3.8	>30	6	−4.5	−4.4	12	−3.0	−2.9	Emphasis to 25:35:40 from low–glycemic index sources
Melanson et al. [32]	59	39.1 ± 7.1	31.1 ± 2.5	3	−3.4 ± 2.8	−4.0	No data			17:36:47 (Base)
										22:33:47 (3 Mo)
**Mediterranean**										
Elhayany et al. [33]	89	56.0 ± 6.1	27–34	No data			12	−7.4	−8.7	Recommend to 20:30:50
Auster et al. [34]	100	52.4 ± 0.9	30.1 ± 0.3	3	−6.1	−7.2	No data			
**Ornish**										
Dansinger et al. [20]	40	49 ± 12	35 ± 3.9	6	−3.6 ± 6.7	−3.5	12	−3.3 ± 7.3	−3.2	18:35:49 (Base)
										17:29:55 (6 Mo)
										17:32:48 (12 Mo)
Gardner et al. [23]	76	42 ± 6	32 ± 3	6	−2.5	−2.9	12	−2.2	−2.6	16:35:48 (Base)
										18:28:53 (6 Mo)
										18:30:52 (12 Mo)
**Paleolithic**										
Mellberg et al. [13]	35	59.5 ± 5.5	32.7 ± 3.6	6	−7.85	−9.0	24	−9.2	−10.6	17:33:46 (Base)
										23:44:29 (6 Mo)
										22:40:34 (24 Mo)
**Zone**										
Dansinger et al. [20]	40	51 ± 9	34 ± 4.5	6	−3.4 ± 5.7	−3.4	12	−3.2 ± 6.0	−3.2	18:35:46 (Base)
										19:32:42 (6 Mo)
										21:37:39 (12 Mo)
McAuley et al. [24]	30	30–70	34.5 ± 5.3	6	−6.9	−7.4				17:31:47 (Base)
										26:35:35 (6 Mo)
Gardner et al. [23]	79	40 ± 6	31 ± 3	6	−2.0	−2.4	12	−1.5	−1.8	17:37:47 (Base)
										20:36:44 (6 Mo)
										20:35:45 (12 Mo)

Mo, months, Base, baseline. Short- and long-term weight change showed mean ± standard deviation (SD). Short-term assessment period is less than six months; long-term is more than 12 months. Age and body mass index (BMI) were shown mean ± SD or range. ^†^ Data combined from two cohorts that underwent the same intervention for either four or six months. * Participants were given the option to continue the diet to which they were allocated for an additional six months (making the total dietary intervention 12 months).

## Supplementary Materials

The following are available online at www.mdpi.com/2072-6643/9/8/822/s1, Table S1: Characteristics of selected articles; Table S2: Assessment on the risk of bias; Figure S1: Summary of study-level risk of bias assessment.

## References

[1] Mann T., Tomiyama A.J., Westling E., Lew A.M., Samuels B., Chatman J. (2007). Medicare’s search for effective obesity treatments: Diets are not the answer. Am. Psychol..

[2] Johnston B.C., Kanters S., Bandayrel K., Wu P., Naji F., Siemieniuk R.A., Ball G.D., Busse J.W., Thorlund K., Guyatt G. (2014). Comparison of weight loss among named diet programs in overweight and obese adults: A meta-analysis. JAMA.

[3] U.S. Department of Agriculture, U.S. Department of Health and Human Services (2010). Dietary Guidelines for Americans.

[4] Cohen E., Cragg M., deFonseka J., Hite A., Rosenberg M., Zhou B. (2015). Statistical review of US macronutrient consumption data, 1965–2011: Americans have been following dietary guidelines, coincident with the rise in obesity. Nutrition.

[5] Makris A., Foster G.D. (2011). Dietary approaches to the treatment of obesity. Psychiatr. Clin. N. Am..

[6] Riley R.E. (1999). Popular weight loss diets. Clin. Sports Med..

[7] Volpe S.L. (2006). Popular weight reduction diets. J. Cardiovasc. Nurses.

[8] Atkins R. (2002). Dr. Atkins’ New Diet Revolution.

[9] Appel L.J., Moore T.J., Obarzanek E., Vollmer W.M., Svetkey L.P., Sacks F.M., Bray G.A., Vogt T.M., Cutler J.A., Windhauser M.M. (1997). A clinical trial of the effects of dietary patterns on blood pressure. N. Engl. J. Med..

[10] Jenkins D.J., Wong J.M., Kendall C.W., Esfahani A., Ng V.W., Leong T.C., Faulkner D.A., Vidgen E., Paul G., Mukherjea R. (2014). Effect of a 6-month vegan low-carbohydrate (‘Eco-Atkins’) diet on cardiovascular risk factors and body weight in hyperlipidaemic adults: A randomised controlled trial. BMJ Open.

[11] Ebbeling C.B., Ludwig D.S., Goran M.I., Sothern M.S. (2005). Dietary approaches for obesity treatment and prevention in children and adolescents. Handbook of Pediatric Obesity: Epidemiology, Etiology and Prevention.

[12] Ornish D. (2001). Eat More, Weigh Less.

[13] Mellberg C., Sandberg S., Ryberg M., Eriksson M., Brage S., Larsson C., Olsson T., Lindahl B. (2014). Long-term effects of a Palaeolithic-type diet in obese postmenopausal women: A 2-year randomized trial. Eur. J. Clin. Nutr..

[14] Turner-McGrievy G.M., Davidson C.R., Wingard E.E., Wilcox S., Frongillo E.A. (2015). Comparative effectiveness of plant-based diets for weight loss: A randomized controlled trial of five different diets. Nutrition.

[15] Sears B., Lawren W. (1995). Enter the Zone.

[16] The Best Diet Rankings 2016—The Best Weight-Loss Diets, U.S. News & World Report L.P.. http://health.usnews.com/best-diet/best-weight-loss-diets.

[17] Donnelly J.E., Blair S.N., Jakicic J.M., Manore M.M., Rankin J.W., Smith B.K. (2009). Appropriate physical activity intervention strategies for weight loss and prevention of weight regain for adults. Med. Sci. Sports Exerc..

[18] Stevens J., Truesdale K.P., McClain J.E., Cai J. (2006). The definition of weight maintenance. Int. J. Obes..

[19] Higgins J.P.T., Green S. (2007). Cochrane Handbook for Systematic Reviews of Interventions, Version 5.1.0.

[20] Dansinger M.L., Gleason J.A., Griffith J.L., Selker H.P., Schaefer E.J. (2005). Comparison of the atkins, ornish, weight watchers, and zone diets for weight loss and heart disease risk reduction: A randomized trial. JAMA.

[21] Foster G.D., Wyatt H.R., Hill J.O., McGuckin B.G., Brill C., Mohammed B.S., Szapary P.O., Rader D.J., Edman J.S., Klein S. (2003). A randomized trial of a low-carbohydrate diet for obesity. N. Engl. J. Med..

[22] Foster G.D., Wyatt H.R., Hill J.O., Makris A.P., Rosenbaum D.L., Brill C., Stein R.I., Mohammed B.S., Miller B., Rader D.J. (2010). Weight and metabolic outcomes after 2 years on a low-carbohydrate versus low-fat diet: A randomized trial. Ann. Int. Med..

[23] Gardner C.D., Kiazand A., Alhassan S., Kim S., Stafford R.S., Balise R.R., Kraemer H.C., King A.C. (2007). Comparison of the Atkins, Zone, Ornish, and LEARN diets for change in weight and related risk factors among overweight premenopausal women: The A TO Z Weight Loss Study: A randomized trial. JAMA.

[24] McAuley K.A., Hopkins C.M., Smith K.J., McLay R.T., Williams S.M., Taylor R.W., Mann J.I. (2005). Comparison of high-fat and high-protein diets with a high-carbohydrate diet in insulin-resistant obese women. Diabetologia.

[25] Summer S.S., Brehm B.J., Benoit S.C., D’Alessio D.A. (2011). Adiponectin changes in relation to the macronutrient composition of a weight-loss diet. Obesity.

[26] Truby H., Baic S., de Looy A., Fox K.R., Livingstone M.B., Logan C.M., Macdonald I.A., Morgan L.M., Taylor M.A., Millward D.J. (2006). Randomised controlled trial of four commercial weight loss programmes in the UK: Initial findings from the BBC “diet trials”. BMJ.

[27] Yancy W.S., Westman E.C., McDuffie J.R., Grambow S.C., Jeffreys A.S., Bolton J., Chalecki A., Oddone E.Z. (2010). A randomized trial of a low-carbohydrate diet vs. orlistat plus a low-fat diet for weight loss. Arch. Int. Med..

[28] Shai I., Schwarzfuchs D., Henkin Y., Shahar D.R., Witkow S., Greenberg I., Golan R., Fraser D., Bolotin A., Vardi H. (2008). Weight loss with a low-carbohydrate, Mediterranean, or low-fat diet. N. Engl. J. Med..

[29] Davis N.J., Tomuta N., Schechter C., Isasi C.R., Segal-Isaacson C.J., Stein D., Zonszein J., Wylie-Rosett J. (2009). Comparative study of the effects of a 1-year dietary intervention of a low-carbohydrate diet versus a low-fat diet on weight and glycemic control in type 2 diabetes. Diabetes Care.

[30] Blumenthal J.A., Babyak M.A., Sherwood A., Craighead L., Lin P.H., Johnson J., Watkins L.L., Wang J.T., Kuhn C., Feinglos M. (2010). Effects of the dietary approaches to stop hypertension diet alone and in combination with exercise and caloric restriction on insulin sensitivity and lipids. Hypertension.

[31] Ebbeling C.B., Leidig M.M., Feldman H.A., Lovesky M.M., Ludwig D.S. (2007). Effects of a low-glycemic load vs low-fat diet in obese young adults: A randomized trial. JAMA.

[32] Melanson K.J., Summers A., Nguyen V., Brosnahan J., Lowndes J., Angelopoulos T.J., Rippe J.M. (2012). Body composition, dietary composition, and components of metabolic syndrome in overweight and obese adults after a 12-week trial on dietary treatments focused on portion control, energy density, or glycemic index. Nutr. J..

[33] Elhayany A., Lustman A., Abel R., Attal-Singer J., Vinker S. (2010). A low carbohydrate Mediterranean diet improves cardiovascular risk factors and diabetes control among overweight patients with type 2 diabetes mellitus: A 1-year prospective randomized intervention study. Diabetes Obes. Metab..

[34] Austel A., Ranke C., Wagner N., Gorge J., Ellrott T. (2015). Weight loss with a modified Mediterranean-type diet using fat modification: A randomized controlled trial. Eur. J. Clin. Nutr..

[35] Dietary Guidelines Advisory Committee (2015). Scientific Report of the 2015 Dietary Guidelines Advisory Committee: Advisory Report to the Secretary of Health and Human Services and the Secretary of Agriculture.

[36] Chen T.Y., Smith W., Rosenstock J.L., Lessnau K.D. (2006). A life-threatening complication of Atkins diet. Lancet.

[37] Expert Panel on the Identification, Evaluation, and Treatment of Overweight in Adults (1998). Clinical guidelines on the identification, evaluation, and treatment of overweight and obesity in adults: Executive summary. Am. J. Clin. Nutr..

[38] Pi-Sunyer F.X. (1996). A review of long-term studies evaluating the efficacy of weight loss in ameliorating disorders associated with obesity. Clin. Ther..

[39] Anton S.D., Foreyt J., Perri M.G. (2014). Preventing Weight Regain after Weight Loss. Handbook of Obesity Treatment: Clinical Applications.

[40] Scheen A.J. (2008). The future of obesity: New drugs versus lifestyle interventions. Expert Opin. Investig. Drugs.

[41] Noakes T.D., Windt J. (2017). Evidence that supports the prescription of low-carbohydrate high-fat diets: A narrative review. Br. J. Sports Med..

[42] Ebbeling C.B., Swain J.F., Feldman H.A., Wong W.W., Hachey D.L., Garcia-Lago E., Ludwig D.S. (2012). Effects of dietary composition on energy expenditure during weight-loss maintenance. JAMA.

[43] Rolland Y., Czerwinski S., van Abellan K.G., Morley J.E., Cesari M., Onder G., Woo J., Baumgartner R., Pillard F., Boirie Y. (2008). Sarcopenia: Its assessment, etiology, pathogenesis, consequences and future perspectives. J. Nutr. Health Aging.

[44] Zamboni M., Rossi A.P., Fantin F., Zamboni G., Chirumbolo S., Zoico E., Mazzali G. (2014). Adipose tissue, diet and aging. Mech. Ageing Dev..

[45] Romero-Corral A., Somers V.K., Sierra-Johnson J., Korenfeld Y., Boarin S., Korinek J., Jensen M.D., Parati G., Lopez-Jimenez F. (2010). Normal weight obesity: A risk factor for cardiometabolic dysregulation and cardiovascular mortality. Eur. Heart J..

[46] Oliveros E., Somers V.K., Sochor O., Goel K., Lopez-Jimenez F. (2014). The concept of normal weight obesity. Prog. Cardiovasc. Dis..

[47] Marques-Vidal P., Pecoud A., Hayoz D., Paccaud F., Mooser V., Waeber G., Vollenweider P. (2010). Normal weight obesity: Relationship with lipids, glycaemic status, liver enzymes and inflammation. Nutr. Metab. Cardiovasc. Dis..

[48] Kosmala W., Jedrzejuk D., Derzhko R., Przewlocka-Kosmala M., Mysiak A., Bednarek-Tupikowska G. (2012). Left ventricular function impairment in patients with normal-weight obesity: Contribution of abdominal fat deposition, profibrotic state, reduced insulin sensitivity, and proinflammatory activation. Circ. Cardiovasc. Imaging.

[49] Batsis J.A., Sahakyan K.R., Rodriguez-Escudero J.P., Bartels S.J., Somers V.K., Lopez-Jimenez F. (2013). Normal weight obesity and mortality in United States subjects ≥60 years of age (from the Third National Health and Nutrition Examination Survey). Am. J. Cardiol..

