# Supplementary material for: Effects of Popular Diets without Specific Calorie Targets on Weight Loss Outcomes: Systematic Review of Findings from Clinical Trials
Source: Nutrients. 2017 Jul 31;9(8):822. doi: 10.3390/nu9080822 (PMC5579615; doi:10.3390/nu9080822)
Supplement: Supplementary file 1 [file nutrients-09-00822-s001.zip › nutrients-204192-Supplemental Material.pdf]

Online Supplemental Material

Effects of Popular Diets Without Specific Calorie Targets on Weight Loss Outcomes: Systematic Review of Findings from Clinical Trials

Stephen D. Anton, Ph.D.,[1,2 *] Azumi Hida, Ph.D.,[1,3 *] Kacey Heekin, B.A.,[1] Kristen Sowalsky, D.C.,[4] Christy Karabetian, M.S.,[1,2] Heather Mutchie, B.A.,[1,5] Christiaan Leeuwenburgh, Ph.D.,[1] Todd M. Manini, Ph.D.,[1] & Tracey E. Barnett, Ph.D.[6]

[*] Contributed equally

Search strategy

Relevant articles, published before 30 September 2016, were identified through three electronic databases (PubMed, Cochrane Library (which included Cochrane Central Register of Controlled Trials (CENTRAL), Cochrane Database of Systematic Reviews (CDSR) and other reviews), and Web of Science). The following search terms were used.

Cochrane Library (Search Manager)

((weight AND diet) AND (overweight OR obesity) AND (clinical trial) AND (each diet name))

Web of Science (Advanced Search)*

((TS=(weight AND diet) AND TS=(overweight OR obesity)) NOT TS=(rat OR mouse)) AND TS=(clinical trial) *AND* TS=(each diet name) AND English AND Article

*TS means Topic.

Estimated standard deviation

A number of studies reported only mean initial and mean final values in control and intervention groups with corresponding standard deviation. The mean change was calculated as the difference between mean final and mean initial, and the standard deviation as :

The square root of [(SDpre)ˆ2 + (SDpost)ˆ2 - 2r(SDpre*SDpost)], where SDpre was the standard deviation of the mean baseline outcome, SDpost was the standard deviation of the mean follow-up outcome, and r was the correlation between the baseline and follow-up values.

Table S1. Characteristics of selected articles.

Author, year	n	Women (%)	Completed n (%)	Age (mean ±SD) (y)	Duration	Forest plot (Mo)	BMI (Baseline mean±SD or range) (kg/m^2)	Weight (Baseline mean±SD) (kg)
**Atkins diet**								
Foster, 2003,	33	64	20 (61)	44±9.4	3,6,12 Mo	6, 12	33.9±3.8	98.7±19.5
Dansinger, 2005	40	53	21 (53)	47±12	1,2,6,12 Mo	6, 12	35±3.5, 27-42	100±14
McAuley, 2005	31	100	28(90)	45±7.4, 30-70	8,16,24w	6	36.0±3.9, >27	96±10.8
Truby, 2006	57	74	9 (16)	40.9±9.7, 18-65	2,6 Mo	6	31.9±2.2, 27-40	90.3±12.7
Gardner, 2007	77	100	68 (88)	42±5, 25-50	2,6,12 Mo	6, 12	32±4, 27-40	86±13
Shai, 2008	109	9	85 (78)	52±7, 40-65	6, 12, 24 Mo	6, 12	30.8±3.5, >27	91.8±14.3
Davis, 2009	55	82	47 (85)	54±6, >18	3, 6, 12 Mo	6, 12	35±6, ≥25	93.6±18

Foster, 2010	153	67	68 (58)	46.2±9.2, 18-65	3, 6, 12 , 24 Mo	6, 12	36.1±3.6, 30-40	103.3±15.5
Yancy, 2010	72	28	57 (79)	52.9±10.2, 18-70	24, 48 w	6, 12	39.9±6.9, ≥27	123.1±25.4
Summer, 2011	42	100	39 (93)	44.5±9.1, ≥18	6 Mo	6	33.2±2.6, 30-35	90.4±9.1
**DASH diet**								
Blumenthal, 2010,	46	63	44 (96)	51.8±10 ≥35	4 Mo	4	32.8±3.4, 25-40	93.0±14
**Glycemic Index diet**								
Ebbeling, 2007	36	81	28 (78)	28.2±3.8, 18-35	6,12,18 Mo	6, 12	≥30	103.5±17.3
Melanson, 2012	59	88	-	39.1±7.1, 25-50	12 w	3	31.1±2.5, 27-35	84.3±12.4
**Mediterranean diet**								
Elhayany, 2010	89	44	63 (71)	57.4±6.1 30-65	3,6,9,12 Mo	12	31.1±2.8, 27-34	85.5±10.6
Austel, 2015	100	79	72 (72)	52.4±0.9	12 w	3	30.1±0.3	85.1±1.2
**Ornish diet**								
Dansinger, 2005	40	43	20 (50)	49±12	1,2,6,12 Mo	6, 12	35±3.9	103±15
Gardner, 2007	76	100	59 (78)	42±6, 25-50	2,6,12 Mo	6, 12	32±3, 27-40	86±10
**Paleolithic diet**								
Mellberg, 2014	35	100	27 (77)	59.5±5.5	6,24 Mo	6, 24	32.7±3.6, ≥27	87.0±10.6
**Zone diet**								
Dansinger, 2005	40	50	26 (65)	51±9	1,2,6,12 Mo	6, 12	34±4.5	99±18

McAuley, 2005	30	100	29 (97)	47±7.9, 30-70	24w	6	34.5±5.3 >27	93.2±14.5
Gardner, 2007	79	100	61 (77)	40±6, 25-50	2,6,12 Mo	6, 12	31±3, 27-40	84±12

w=weeks, Mo=month.

Table S2. Assessment on the risk of bias.

Author (year)	Random sequence generation (selection bias)	Allocation concealment (selection bias)	Binding of participants and personnel (performance bias)	Binding of outcome assessment (detection bias)	Incomplete outcome data (attrition bias)	Selective reporting (reporting bias)	Other bias (control diet)	Diet group
Foster, 2003	Low	Low	Unclear	Low	Low	Low	High	Atkins, and High-carbohydrate low-fat energy-deficit Conventional diet
Dansinger, 2005	Low	Low	Low	Low	Low	Low	High	Atkins, Ornish, Zone, and Weight Watchers diet
McAuley, 2005	Low	Low	Unclear	Low	Unclear	Low	High	Atkins, Zone, and Conventional High-carbohydrate high-fiber diet
Truby, 2006	Low	Low	High	Low	Low	Low	Low	Atkins, and Control diet
Gardner, 2007	Low	Low	Low	Low	Low	Low	High	Atkins, Ornish, Zone, and LEARN (exercise included) diet
Shai, 2008	Low	Low	Unclear	Low	Low	Low	High	Atkins, Modified Mediterranean, and Low-fat diet (as Reference diet).
Davis, 2009	Low	Low	Unclear	Low	Unclear	Unclear	High	Atkins, and Low-fat diet
Foster, 2010	Low	Low	Unclear	Low	Low	Low	High	Atkins, and Low-fat diet

Yancy, 2010	Low	Low	Unclear	Low	Unclear	Unclear	High	Atkins, and Atkins (calorie restricted) + Orlistat
Summer, 2011	Low	Low	Unclear	Low	Unclear	Unclear	High	Atkins, and Low-fat diet
Blumenthal, 2010	Low	Low	Unclear	Low	Low	Low	Low	DASH, DASH-weight Management (CR and Ex) diet, and Usual care
Ebbeling, 2007	Low	Low	Unclear	Low	Low	Low	High	Glycemic Index, and Low-fat diet
Melanson, 2012	Low	Low	Unclear	Low	Unclear	Unclear	High	Glycemic Index, and Low energy density, and Portion control diet
Elhayany, 2010	Low	Low	Unclear	Low	Unclear	Unclear	Low	Mediterranean, ADA, and Low-carbohydrate Mediterranean diet
Austel, 2015	Low	Low	Unclear	Low	Low	Low	Low	Mediterranean, and Control diet (waiting list)
Mellberg, 2014	Low	Low	Unclear	Low	Low	Low	Low	Paleolithic, and Nordic Nutrition Recommendations (NNR) diet

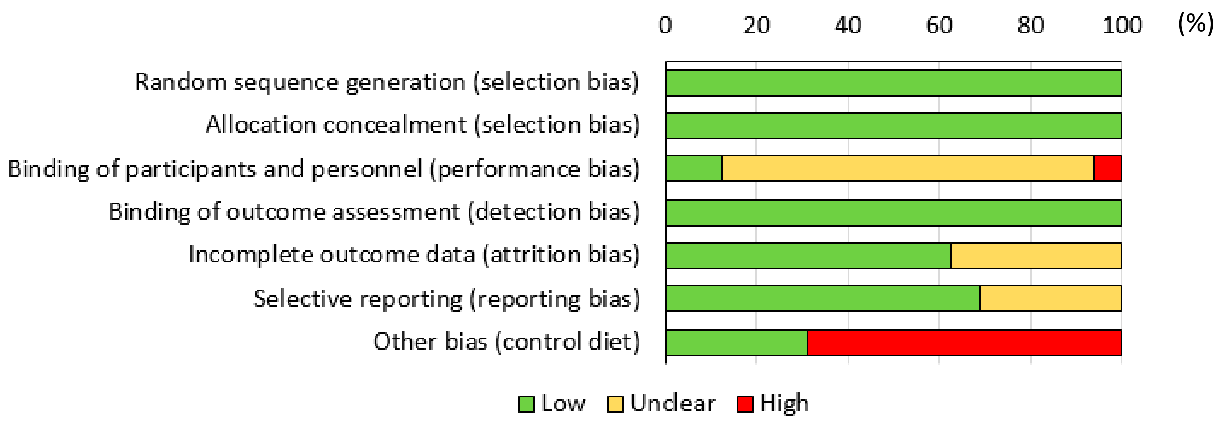

Figure S1. Summary of study-level risk of bias assessment.
